# Planning for your CANOE (Circumspect Awareness and Navigation of Outcomes and Expectations) journey in community-engaged research with Indigenous communities

**DOI:** 10.1016/S2214-109X(25)00262-1

**Published:** 2025-09

**Authors:** Katherine A Collins, Kimberly R Huyser, Michelle Johnson-Jennings

**Affiliations:** Department of Psychology and Health Studies, University of Saskatchewan, Saskatoon, SK, Canada; Department of Sociology, University of British Columbia, Vancouver, BC, Canada; Indigenous Wellness Research Institute, School of Public Health and Social Work, University of Washington, WA, USA

## Abstract

Community engagement has long been recognised as necessary for working with Indigenous communities. Although many researchers are excited to engage with communities and many articles describe the process of community engagement in research, almost none have addressed the foundational question of whether researchers should engage with Indigenous communities for research. In this Viewpoint, we will discuss the Circumspect Awareness and Navigation of Outcomes and Expectations (CANOE) approach, which describes what should be considered before embarking on a community-engaged research journey with Indigenous communities. We build on existing literature regarding understanding the need to recognise positionality, practise reflexivity, assess personal strengths and weaknesses, and consider abilities and skills that can be offered or promised to Indigenous partners. Our goal is to provide principles of being reflexive, intentional, and careful before launching into research with Indigenous communities. Drawing from our combined decades of experience as Indigenous, community-engaged scientists leading national and international community projects, we draw from the extant literature and lessons learned in the field to provide a guiding CANOE approach for community-engaged research. This Viewpoint provides researchers interested in community-engaged projects with the information they need to consider before embarking on their research journey. We provide a set of CANOE self-assessment questions designed to evaluate a researcher’s preparedness, suitability to invest in a research partnership, and adaptability to navigate a research journey with Indigenous communities. Not only should relationships be properly developed and nurtured, but researchers need to fundamentally understand their ability to develop research partnerships that prioritise Indigenous cultural worldviews and protocols in research design, development, testing, and implementation.

## Introduction

Indigenous communities have long requested to be involved and consulted with before, during, and after research activities commence.^1–4^ They have also largely argued for the development of community and academic research partnerships that prioritise their cultural worldviews and protocols in the research design, development, testing, and implementation.^1–4^ Research grounded in and centring Indigenous knowledges and partnerships provides vital infrastructure for communities to address their own health needs, advancing both Indigenous wellbeing and broader scientific understanding. Unfortunately, history is limited for research that involves equal partnerships with Indigenous peoples and communities.^5–8^ Community-based and academic researchers have increasingly recognised and advocated for the validity and significance of Indigenous community-engaged research.^3,9–12^ Despite growing interest, minimal guidance exists on how academic researchers can prepare for Indigenous community-engaged research. We seek to advance the literature by underscoring methodological approaches and the researcher’s readiness to engage in respectful, reciprocal partnerships with Indigenous communities.

Over the past two decades, the landscape of Indigenous research funding has shifted substantially. Academic institutions and funding agencies have increasingly supported Indigenous community-engaged research by issuing targeted calls, adapting funding mechanisms to recognise Indigenous peoples as principal investigators, and affirming the rigour and validity of Indigenous methodologies.^5,13^ More recently, countries with shared histories of settler colonialism, such as Canada and the USA, have launched federal initiatives to promote collaboration with Indigenous peoples and communities (eg, Truth and Reconciliation Commission of Canada: Calls to Action and the National Institutes of Health All of Us Tribal Engagement).^14,15^ In Canada, the three federal research funding agencies have created a joint strategic plan to support Indigenous research capacity.^16^ These agencies are allocating growing resources to Indigenous health research through various mechanisms, including targeted funding calls, dedicated funding allocations—such as the Canadian Institutes for Health Research’s goal of directing 4·6% of its total disbursements to Indigenous health research (achieved in 2021–22)—and the establishment of Indigenous advisory councils and regional engagement initiatives to ensure appropriate oversight and guidance.^16^ Although Indigenous communities receive support for community-engaged activities, concerns continue to plague the field around superficial engagement, scholarly ethics, intent, and long-term sustainability.^5,8,17,18^ Previous research has not addressed the cultural and individual considerations involved in determining whether a researcher should, or when they might be ready to, engage in research with Indigenous communities.

Many Indigenous academic researchers pursue advanced degrees with the goal of serving their communities; however, their numbers remain low due to persistent career barriers rooted in intergenerational trauma and systemic oppression. Meanwhile, increased funding and research opportunities have led to a growing interest among non-Indigenous academic researchers, many of whom are newly aware of health disparities affecting Indigenous communities, in engaging with these populations. This interest is often driven by enthusiasm for the communities themselves, availability of funding, and a perceived desire to contribute or help. Although these funding approaches have spurred increased Indigenous research, many scholars continue to engage with Indigenous communities with minimal preparation or critical reflection, which can perpetuate harm and erode community trust, both in their work and in future research efforts.^11,19–25^ As Indigenous research scientists with over 30 years of combined experience in community-engaged research, we have identified key considerations that should be addressed before initiating research with Indigenous communities. We use the metaphor of a canoe to represent Circumspect Awareness and Navigation of Outcomes and Expectations (CANOE), a framework for guiding ethical and effective engagement with Indigenous partners. Before the journey (ie, engagement with Indigenous communities), researchers must plan, prepare, and understand the social, political, cultural, and physical terrain. Furthermore, community-engaged research requires a philosophical transformation for researchers with perspectives based only on academic training and experiences.

We present an overview of the CANOE approach for community-engaged research with Indigenous partners, grounded in Indigenous cultural considerations and combined with experiences with community partners. We agree with Wallerstein and Duran^11^ that community-engaged research is not a method, but an approach or orientation. Hence, we describe the transformation of thinking required for community-engaged research by detailing how researchers can plan the journey.

## CANOE: framework

There is extensive research on why and how to do all stages of community-engaged research, from choosing an epistemology^6,26^ to forming ethical relationships,^1^ and publishing results.^7^ However, self-reflexive and relational elements of the planning process are often neglected in current approaches to community engagement. There is little to no discussion about how and when to initiate engagement with community partners. Additionally, little guidance exists to help individual researchers assess their own emotional and spiritual readiness to invest the necessary time and effort required to partner with Indigenous communities.

Considering the gap in Indigenous research approaches, we propose our metaphor for community-engaged research as a canoe journey within Indigenous waters. Research partnerships typically include an academic team and a community team. In this analogy, each team enters the river in their canoe from opposite sides, representing their contrasting perspectives and experiences. However, the research team should remain aware that the river belongs to the community, and they can only enter as guests, and only when invited, following proper protocols. In this scenario, the Indigenous cultural knowledge and protocols take precedence, although western science (the common approach in PhD granting universities) could supplement as appropriate and as invited. Ideally, both canoes enter the river at the same place, with the same expectations, and journey up the river while maintaining the same pace with the same destination. This journey is seen as an upriver voyage, going from a place of wonder and forging a path together to reach new ground. The journey requires sustained, consistent effort by both parties. Each team paddles their own canoe, but they should remember that the Indigenous community has sovereignty over all land and waters at all times. Teams should coordinate across the river to keep at the same pace. If they become desynchronised, they can disembark or choose to take a different path as needed. In a successful partnership, both teams make it safely to the agreed-upon destination, each benefiting clearly and transparently, and alerting one another should they no longer mutually benefit.

“Those who have one foot in the canoe, and one foot on the shore, are going to fall into the river.”

Tuscarora proverb

The aforementioned proverb emphasises the full commitment required for community-engaged research with Indigenous peoples. The endeavour is all or nothing; in a real canoe journey, one cannot succeed without both feet firmly situated within the canoe. If one has poor footing, is unprepared, or is not fully committed, then the journey is likely to be short and end with the team being washed away by the river. Similarly, researchers should be fully prepared and invested in engaging in Indigenous community-engaged research, lest they metaphorically capsize. Poorly planned research has been done for decades within Indigenous lands and rivers. However, as with many canoe journeys, individuals should exercise circumspection, or vigilance of self, relationships, and intentions, and the outcomes and expectations associated with such a journey. As they move forward, they should ask themselves: do I need a canoe trip? To where? With whom? Was I properly invited? Furthermore, do I have the proper footing to engage with Indigenous communities in research?

## What type of canoe are you?

Engaging in community-based research with Indigenous communities requires an understanding of the local context, territory, and potential challenges. Much like preparing for a multiday canoe journey, one should first become familiar with the landscape to navigate it with care, skill, and respect. Someone embarking on a canoe journey would assess their canoe’s ability to weather storms, manage turbulent waters, or endure a lengthy journey. They might anticipate challenges that could arise or on-the-ground changes to their plan and strategise on how to cope with them. Community-engaged research involves a dedicated investment of time and effort to establish genuine relationships that do not end when grant funds are spent or the workday ends. Community partners should have real involvement and oversight at every stage of the research process: design, approach, methods, data analysis, results, conclusions, and dissemination.

In the CANOE approach, we ask researchers to take stock of their vessel for research—ie, themselves. The goal is to understand your own strengths and weaknesses and how that affects what you can offer or promise to communities. We outline a series of questions to assist researchers with this understanding (panel 1).

In our experience, opportunities to collaborate with Indigenous communities have a range of approaches and expectations; thus, the individual should recognise their positionality and adaptability before engaging with Indigenous community projects. By reflecting on the CANOE questions, the researcher can identify areas of adaptability and rigidity, informing their approach to relationship building with Indigenous communities. If the researcher has identified any questions that they answered high on the unlikely or unwilling end of the answer scale, the researcher needs to use these as guideposts for their research boundaries and expectations. If most of their answers are in the unwilling or unlikely categories, they might want to re-examine whether they should proceed with community-engaged work, as working with Indigenous communities does call for adaptability and flexibility. If the researcher has identified questions answered at the very likely end of the spectrum, these indicate areas where they might be particularly well suited to meet community needs and expectations directly.

We have created this approach with non-Indigenous researchers in mind; however, it might also serve as a useful starting point for Indigenous researchers who wish to do meaningful, transformative, and beneficial research in their own or other Indigenous communities. Indigenous scholars are often trained primarily in western research frameworks due to the persistent marginalisation of Indigenous Knowledges within educational institutions and broader structures of settler colonial states. This minimal exposure underscores the value of reflective tools such as CANOE for self-assessing readiness to engage in research grounded in Indigenous ways of knowing. However, due to their positionality, Indigenous researchers will need to confront and reflect on additional complexities that are not explicitly covered here. These could include reflecting on being a partial outsider to experiences that might be commonplace for members of their own community and an outsider to other Indigenous communities, along with the cultural humility and inexpertise entailed. Indigenous scholars have a greater responsibility than have non-Indigenous researchers and thus are at a greater risk of harming communities, particularly because they might be more easily trusted by their communities in some circumstances. Therefore, engaging with intentionality, reflexivity, and caution, as indicated by the CANOE approach, is of utmost importance.

As there is a range of answers to the CANOE questions, there is also a range of opportunities to partner and collaborate with Indigenous communities. Based on our collective experiences with our work, there are a few general types of partnerships with Indigenous communities, and we have listed project descriptions ranging from those that are highly prescribed and researcher-driven, to those that are community-generated and co-created with the researcher (panel 2). This list is not an exhaustive or mutually exclusive categorisation but will give prospective researchers a sense of the range that exists and how it might line up with their answers from the CANOE questions.

Although there is much variability and the specific journey of each project can differ, a researcher must regularly practise the CANOE approach, which involves a circumspect awareness of their own positionality, abilities, limitations, expectations, and role within the project, including responsibilities and boundaries.

## Implications of the CANOE approach

In our experience, community-engaged work with Indigenous peoples and communities is highly rewarding. It also requires investment on both personal and professional fronts to conduct transformative research with Indigenous communities. Although we believe that the core principles of the CANOE approach—being reflexive, intentional, and careful—will serve any researcher interested in community engagement, there is incredible diversity among Indigenous populations worldwide and their unique historical and current sociopolitical contexts. This approach serves as a starting point and might not be transferable beyond the context in which it was developed—ie, settler colonial states in North America. Nonetheless, this Viewpoint can serve as an initial framework to consider and adapt as relevant to other Indigenous community research partnerships worldwide.

As outlined in the community engagement literature, one of the motivations for community-engaged research is to dismantle the power relationship where researchers are considered the sole experts and to instead shift power to the community by centring their strengths and expertise while using multiple levels of engagement. We agree with this goal of disrupting power in research. However, with Indigenous peoples and communities, there is an added need to not only recognise the expertise of community members, with cultural and epistemic humility, openness, and receptivity to feedback and redirection, but also to acknowledge the role of Indigenous sovereignty in research and over any data generated. Sovereignty means that Indigenous communities have an inherent right to exercise authority over each stage of the research process and have complete ownership over all outcomes and any data. This means that shifting power in research to Indigenous communities is not merely a hoped-for goal, but a necessity.

## Panel 1: What kind of waters is your canoe prepared to navigate?

### Self-assessment questions: scaled from very unlikely to very likely or very unwilling to very willing

#### Circumspect awareness

Involves carefully considering all circumstances and possible consequences, while continually assessing the terrain and being aware of relevant issues and concerns.

- How willing are you to do the difficult work of preparation (eg, learning about the history of the community, relations between university and community, current and historical wrongs, ethics track record, culture, customs, etc) that is required?
- How likely are you to develop genuine relationships with community members?
- How likely are you to relinquish academic freedoms when they clash with community priorities?
- How well do you understand subtle social cues from people?
- How likely are you to commit to a long-term project or relationship without grant funds?
- How willing are you to put in more effort under more oversight?

#### Navigation

Navigating the partnership with Indigenous communities involves moving through preconceptions and other challenges.

- How likely are you to weather stormy conditions and navigate them safely?
- How willing are you to gain the skills to navigate turbulent waters (eg, difficult or sensitive situations)?
- How likely are you to be resilient enough to last many years of involvement across various situations?
- How likely are you to navigate the waters of community-engaged research without causing ecological destruction?
- How well can you bounce back from criticism or correction?
- How well can you compromise when your plans are challenged?
- How well do you handle unexpected events? How likely are you to describe yourself as ready for anything?
- How capable are you of being respectful and recognising the sacrifice of community partners?
- How willing are you to recognise and be respectful of the effort of community partners to guide you through waters or territory you are unfamiliar with?
- How likely are you to respect those with life experiences, viewpoints, and training that differs from your own?

#### Outcomes

There could be various and possibly differing outcomes of the research project and partnership with the Indigenous community that require skilful manoeuvring.

- How likely are you to accept different outcomes than you expected and planned for? Would it be okay with you if the community decided not to publish or present from the project?
- How likely are you to dedicate years to a community project knowing you might not achieve the typical academic rewards?
- How likely are you to prioritise social and health equity, human rights, and social justice over other values in your research project and partnership?
- How likely are you to give up your research interests to respond to community needs and priorities? Is the topic of research a deal breaker?

#### Expectations

Expectations of how community-engaged research unfolds for the researcher and the Indigenous community need careful attention.

- How willing are you to shift power to community leads?
- How likely are you to recognise that your experience and knowledge are limited?
- How likely are you to follow the direction of community leaders, Elders, and Knowledge Keepers, even if they contradict your own goals?
- How willing are you to receive course correction (ie, criticism or correction) from community members, Elders, or Knowledge Keepers?
- How likely are you to take on out-of-pocket costs if necessary?
- How willing are you to integrate protocols and expectations of respecting the land, water, and non-human living creatures into your research?
- How willing are you to learn about and practise respect for the land and waters you navigate while in your canoe?

## Panel 2: Examples of partnerships with Indigenous communities

- A researcher joins a pre-existing project and provides specific skills or knowledge to advance the aims of the project
- A researcher has an ongoing project with non-Indigenous communities and there is an Indigenous community joining the existing protocol
- An Indigenous community has a project and has outlined a role and expectations for a researcher collaborator to provide a unique contribution to a project
- The researcher uses community-engaged approaches to identify and develop a project with an Indigenous community while keeping their own research agenda goals integrated into the project
- The researcher uses community-engaged approaches to identify and develop a project with an Indigenous community and centres the community goals and outcomes in the project
